# Improving the Performance of an Auditory Brain-Computer Interface Using Virtual Sound Sources by Shortening Stimulus Onset Asynchrony

**DOI:** 10.3389/fnins.2018.00108

**Published:** 2018-02-27

**Authors:** Miho Sugi, Yutaka Hagimoto, Isao Nambu, Alejandro Gonzalez, Yoshinori Takei, Shohei Yano, Haruhide Hokari, Yasuhiro Wada

**Affiliations:** ^1^Graduate School of Engineering, Nagaoka University of Technology, Nagaoka, Japan; ^2^Department of Electrical and Information Engineering, National Institute of Technology, Akita College, Akita, Japan; ^3^Department of Electrical and Electronic Systems Engineering, National Institute of Technology, Nagaoka College, Nagaoka, Japan

**Keywords:** auditory BCI, EEG, P300, virtual sounds, SOA

## Abstract

Recently, a brain-computer interface (BCI) using virtual sound sources has been proposed for estimating user intention via electroencephalogram (EEG) in an oddball task. However, its performance is still insufficient for practical use. In this study, we examine the impact that shortening the stimulus onset asynchrony (SOA) has on this auditory BCI. While very short SOA might improve its performance, sound perception and task performance become difficult, and event-related potentials (ERPs) may not be induced if the SOA is too short. Therefore, we carried out behavioral and EEG experiments to determine the optimal SOA. In the experiments, participants were instructed to direct attention to one of six virtual sounds (target direction). We used eight different SOA conditions: 200, 300, 400, 500, 600, 700, 800, and 1,100 ms. In the behavioral experiment, we recorded participant behavioral responses to target direction and evaluated recognition performance of the stimuli. In all SOA conditions, recognition accuracy was over 85%, indicating that participants could recognize the target stimuli correctly. Next, using a silent counting task in the EEG experiment, we found significant differences between target and non-target sound directions in all but the 200-ms SOA condition. When we calculated an identification accuracy using Fisher discriminant analysis (FDA), the SOA could be shortened by 400 ms without decreasing the identification accuracies. Thus, improvements in performance (evaluated by BCI utility) could be achieved. On average, higher BCI utilities were obtained in the 400 and 500-ms SOA conditions. Thus, auditory BCI performance can be optimized for both behavioral and neurophysiological responses by shortening the SOA.

## Introduction

Brain-computer interfaces (BCIs)—systems that can operate external devices using only brain signals—have been actively studied in recent years (Wolpaw and Wolpaw, 2012), and are expected to provide a method of communication and interaction for people with severe motor disabilities. Many BCI studies have used event-related potentials (ERPs) (Farwell and Donchin, 1988; Wolpaw and Wolpaw, 2012), which are brain signals that occur in relation to some event. For example, the P300 obtained during the oddball paradigm is a positive ERP that occurs approximately 300 ms after stimulus presentation (Polich, 2007). Although most BCI studies have used P300 features evoked by visual stimuli (Sellers et al., 2006; Martens et al., 2009; Halder et al., 2013), studies using auditory stimuli, such as different types, tones, or directions of sound, have also been reported (Klobassa et al., 2009; Halder et al., 2010, 2013; Kanoh et al., 2010; Kim et al., 2011). Recently, auditory BCIs using spatial information such as sound-source direction have been studied and are considered intuitive and easy to use (Schreuder et al., 2010, 2011; Gao et al., 2011; Käthner et al., 2013; Nambu et al., 2013; Simon et al., 2014). The use of a virtual sound source as a stimulus is thought to have additional advantages (Gao et al., 2011; Käthner et al., 2013; Nambu et al., 2013; Simon et al., 2014). For example, in a previous study (Nambu et al., 2013) we used a system of auditory stimuli from different directions that was generated by out-of-head sound localization technology and presented as virtual sound over earphones (Shimada and Hayashi, 1995). The virtual sounds were produced using individual head-related transfer functions (HRTFs). Because this system can generate spatial sound accurately without having to place loudspeakers, it is considered a viable option for use in a compact and portable BCI system.

Thus, an auditory BCI using virtual sound has great potential for practical applications. However, compared with visual BCIs, which have performed better in past research (as evaluated by information transfer rate or BCI utility), an auditory BCI system using virtual sounds with sufficient performance has yet to be developed (Käthner et al., 2013; Nambu et al., 2013).

In this study, we aimed to improve the performance of an auditory BCI using virtual sound. For this purpose, we focused on manipulating the duration between presented stimuli, usually referred to as stimulus onset asynchrony (SOA). Shortening the SOA is a very effective means of improving BCI performance with a simple change in experimental design (Farwell and Donchin, 1988; Allison and Pineda, 2006; Sellers et al., 2006; McFarland et al., 2011; Höhne and Tangermann, 2012; Lu et al., 2013). These previous studies have suggested a 100–200-ms SOA is applicable for visual BCIs and for a simple auditory BCI. However, it remains unclear whether short SOA is useful for many other settings as well as auditory BCIs using virtual sound.

To improve BCI performance by shortening the SOA, it is necessary to consider several aspects. First, if the SOA is too short, the sound perception might become too difficult, the task might not be performed correctly, and fewer and smaller ERPs might be induced. Indeed, previous ERP studies have shown that P300 is attenuated as task difficulty increases (Kramer et al., 1986; Polich, 1987; Kim et al., 2008). In this case, BCI performance is expected to worsen. Therefore, to improve BCI performance by shortening the SOA of stimulus presentation, the participant needs to be able to recognize the direction from which the stimulus sound comes at the shortened SOA. Additionally, even if participants can recognize the sound direction, whether ERPs can be induced clearly with respect to the attended sound and whether the performance of the auditory BCI can be improved remain unknown. Short target-to-target intervals have been shown to cause small ERPs (Gonsalvez and Polich, 2002; Gonsalvez et al., 2007) because of refractory effects and minimally required attentional effort (Polich, 2007). Because target-to-target interval is closely related to SOA (when target probability and stimulus sequence are fixed), similar low ERPs are likely to be observed when the SOA is too short.

Therefore, the optimal SOA that maximizes BCI performance is the one for which participants can still accomplish the task, and for which sufficient identification accuracy is obtained. For these reasons, determining the optimal SOA is required for assessing both behavioral and electroencephalographic (EEG) data (ERP and identification accuracy) (Allison and Pineda, 2006). Several BCI studies have examined the effects of SOA on ERPs and/or BCI performance in visual (Farwell and Donchin, 1988; Sellers et al., 2006; McFarland et al., 2011; Lu et al., 2013) and auditory BCIs (Höhne and Tangermann, 2012) without considering behavioral data. Further, although a few studies have examined behavioral responses (or count) and ERPs (Gonsalvez and Polich, 2002; Allison and Pineda, 2006), they did not check them in the context of BCI performance. Thus, to our knowledge, no study has yet investigated both behavioral and EEG data with an auditory BCI.

To examine the effectiveness of shortening the SOA on an auditory BCI using virtual sounds, here we conducted both behavioral and EEG measurements using an oddball paradigm. In each experiment, we tested eight different SOA conditions: 200, 300, 400, 500, 600, 700, 800, and 1,100 ms. Using behavioral button-press responses to the pre-defined target direction of the sound, we first checked that the participants recognized the sound direction when the SOA was shortened relative to the 1,100-ms SOA condition that we used in a previous study (Nambu et al., 2013). Then, we examined the ERPs for the target and non-target sub-trials, and calculated the identification accuracies offline using a regularized Fisher discriminant analysis (FDA) (Gonzalez et al., 2014). Finally, we evaluated the BCI performance using BCI utility (Dal Seno et al., 2010). Based on these results, we were able to determine how much the SOA should be shortened.

## Materials and methods

### Participants

This study was conducted according to the Declaration of Helsinki and approved by the ethics board of the Nagaoka University of Technology. Nine healthy people (eight males and one female, mean age: 22.5) participated. All participants were given information about the experiment and then signed consent forms.

### Experiment condition

**Figure 1:**
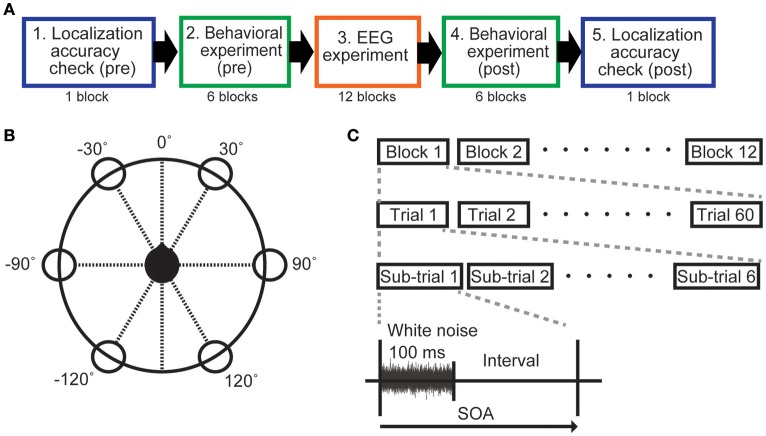
Experimental protocol. **(A)** Outline of the experiments. **(B)** The sound image was located in one of six different directions (30°, −30°, 90°, −90°, 150°, −150°) with 0° directly facing the user (black head) in the horizontal plane. **(C)** A sub-trial comprised a 100-ms sound (white noise) and an interval without sound. The SOA was manipulated by changing the interval. Sound duration was fixed at 100 ms for all SOA conditions. For each experiment, one of the eight SOAs (200, 300, 400, 500, 600, 700, 800, and 1,100 ms) was selected.

All participants carried out eight experimental sets, each of which used one of the eight different SOAs. Each SOA condition was tested on a different day. A single experimental set comprised a localization accuracy check, the behavioral experiment, and the EEG experiment. Experiments were conducted in the following order: localization accuracy check (pre), behavioral experiment (pre), EEG experiment, behavioral experiment (post), and localization accuracy check (post) (Figure 1A).

### Auditory stimuli (virtual sound)

We used virtual sound sources that employed out-of-head sound localization (Shimada and Hayashi, 1995; Nambu et al., 2013) as auditory stimuli. The out-of-head sound localization technology can produce accurate virtual sounds from earphones using the HRTF. This technique can be realized by equalizing the stimulation in the eardrum generated by sound waves from loudspeakers and headphones. The principle of this technique is described by following equation:

(1) $$\begin{array}{r} HRTF=SSTF\cdot LSTF^{-1} \end{array}$$

where *SSTF* (referred to as spatial sound transfer function) is a transfer function from loudspeakers and ear canals. *LSTF* is loudspeaker transfer function that is defined for each loudspeaker (for details, see Shimada and Hayashi, 1995; Yano et al., 2000).

However, sound waves in the eardrum are different for each participant because of the shape of the head and the pinna. Therefore, the HRTFs (SSFTs) need to be measured separately for each user to ensure accurate virtual sound synthesis.

Measurements were made in a sound insulation room that was insulated with sound-absorbing glass wool material. A chair was placed at the center of the room (4.0 × 5.3 m). For HRTF measurements, a miniature microphone (UC-92H, Rion, Japan) was placed at the entrance to the participants' ear canals to measure white noise (100 Hz−15 kHz) that was produced by a set of speakers (SD-0.6, Soundevice, Japan) arranged around them in six different directions (60° apart). The speakers were located 1.5 m from the participants, and the height from the floor to the center of the speakers was set to 1.2 m, which was the same height as the participants' ears. The height and angles of the chair were adjusted so that the position of the small microphone was aligned with the speaker at the 90° position (see Figure 1B). Once everything was adjusted for a participant, we measured the impulse response of the HRTFs. Including preparation time, the total procedure took about total 30–60 min per participant, although actual measurement was completed within one min for each direction. The virtual sounds were produced by convolving the measured impulse responses with white noise. We prepared six different directions for the virtual sound (30°, −30°, 90°, −90°, 150°, −150°), using the same white noise stimulus for each direction.

### Localization-accuracy check

Even though we used HRTFs that were customized for each participant, virtual sound is not always localized well. Practically, localization errors can occur on different days even when the same HRTF is used. To ensure that participants always correctly identified the position of the out-of-head virtual sound, a localization-accuracy check was carried out in all SOA experiments. One check-trial lasted 3,000 ms, including the 100-ms sound stimulus presentation. After stimulus presentation, the participant was asked to verbally report the perceived direction of the stimulus. Sound directions were randomly selected. A single block of the localization-accuracy check was performed before and after the behavioral experiments, respectively (Figure 1A). Each block contained 60 check-trials, and the accuracy of direction identification for the presented sounds was calculated using both blocks.

### Behavioral experiment

#### Procedures

The behavioral experiment was carried out using a button-press task to confirm whether participants could recognize the sound direction in each SOA condition. The sound image could be located in one of six directions described above. We used an oddball experimental paradigm. One direction was defined as the target, and the other directions were non-target. We instructed participants to press the button as soon as possible when a sound was heard from the target direction.

Six blocks of the behavioral experiment were performed block before and after the EEG experiment in each SOA condition. Each of the six directions was the target in one of the six blocks. The time between blocks was about 1 min. Each block comprised 10 trials, and each trial included six sub-trials: one target sub-trial and five non-target sub-trials (Figure 1C). In each sub-trial, the white noise stimulus was presented for 100 ms followed by a silent interval. SOA was defined as the duration of the white noise plus the silent interval (Figure 1C), and varied by condition (200, 300, 400, 500, 600, 700, 800, and 1,100 ms). In the same way, as in the localization accuracy check, the direction of sounds in each sub-trial was pseudorandomized. Note that definition of a trial here differs from the check-trial used in the localization check (see above).

#### Analysis

The behavioral responses in the button-press task were analyzed for eight out of nine participants. One participant was excluded from the analysis because of missing data. Using data obtained in both pre- and post-behavioral experiments, we obtained target recognition accuracy, which was calculated by checking whether the participant pressed the button after the presentation of the target sound. We defined a correct button press as one that occurred in a time window starting at 150 ms after the beginning of sound presentation and lasting until the next sound presentation. This definition was based on the assumptions that: (1) motor response latency to auditory stimuli is probably at least 160 ms (Welford, 1988); and (2) they finished their responses before the next sound. However, for very short SOAs, the second assumption does not hold because they might be shorter than the participant's reaction time (RT). In fact, when we checked the average RTs across participants for short SOA conditions, they were 400–500 ms. Therefore, we counted responses within the range of 150–600 ms after the stimulus for short SOA conditions (<500 ms).

### EEG experiment

#### Procedures

The EEG experiment used basically the same oddball task as the behavioral experiment. However, instead of the button press, we instructed the participants to silently count the number of times that the stimulus sound was heard from the target direction (referred to as the count task). Each of eight experimental sets used one of the eight SOAs (200, 300, 400, 500, 600, 700, 800, and 1,100 ms). Each experiment comprised 12 blocks, and each block included 30 ± 1 trials. As in the behavioral experiment, single trials consisted of six sub-trials (Figure 1C). A sub-trial consisted of 100 ms of white noise followed by the variable silent interval, in which no sound was produced. The target direction was relocated for each block, and each direction served as a target twice in each SOA condition (total 12 blocks). After completing six blocks, we took a break for about 5 min.

We measured the EEG signals using a digital electroencephalograph system (Active Two, BioSemi, Amsterdam, The Netherlands) with 64 electrodes attached to the scalp using a cap. The driven right leg passive electrode and common mode sense active electrode were attached to the left and right ears, respectively, to reduce impedance (for details, see^1^). The EEG data were sampled at 256 Hz. We instructed the participants to perform the task with their eyes closed.

### Preprocessing

Preprocessing of EEG data was performed for all SOA conditions. Data were high-pass and low-pass filtered. The low-pass cutoff frequency was 0.1 Hz and the high-pass cutoff frequency was 8 Hz (Gonzalez et al., 2014). After the filtering, we removed motion artifacts using the ADJUST toolbox (Mognon et al., 2011). We used a 1,000-ms span of EEG data to determine the identification accuracy for each different SOA. For each channel, we averaged the last 100 ms of the signal before stimulus onset as a baseline correction, and subtracted it from the measured data. Then we downsampled the data by averaging every 10 samples.

### ERP analysis

For each SOA condition, we examined differences in ERP waveforms to check whether they were evoked by sound from the target direction. Data from each participant were averaged for each target and non-target waveform in each SOA condition.

First, we examined the temporal changes in the ERP at the Pz channel. Significant differences were tested for using a paired *t*-test for each time sample in the interval of 0–1,000 ms. To eliminate false positives caused by multiple comparison, the FDR (False Discovery Rate) was adjusted by a method proposed by Benjamini and Hochberg (1995) (number of samples *N*_*time*_ = 256).

**Figure 2:**
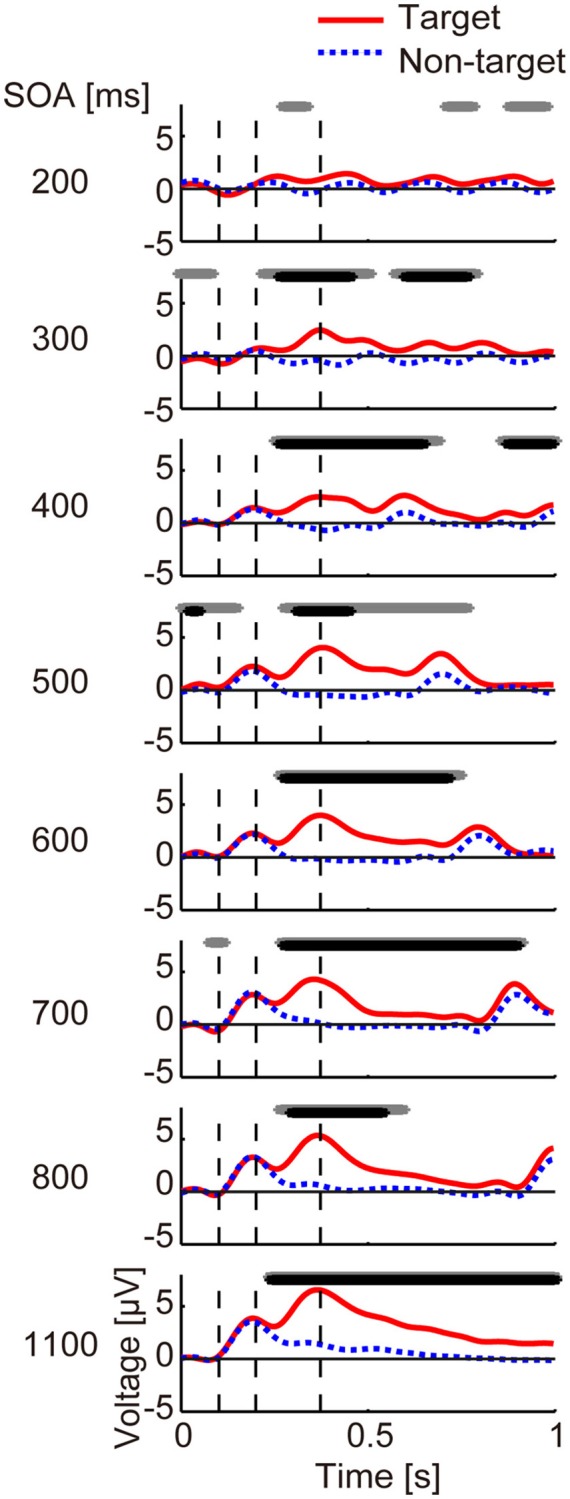
Averaged ERP data for each SOA. Averaged ERPs across participants at channel Pz from 0 to 1 s. The red line shows target data and the blue dashed line shows non-target data. The black and gray lines at the top of each panel represent duration, showing a significant difference between target and non-target data using paired *t*-tests (*p* < 0.05) with and without FDR correction, respectively. Vertical dashed lines show the averaged latencies (98, 203, and 375 ms; average across SOA conditions) at which positive or negative peaks occurred, each of which probably represents N100, P200, and P300, respectively.

Next, we examined spatiotemporal ERP differences between the target and non-target sub-trials, focusing on differences in ERP data at the three times points at which we expected to see N100, P200, or P300 (Allison and Pineda, 2006). Based on previous studies (Gonsalvez and Polich, 2002; Allison and Pineda, 2006), we determined the peak onsets (i.e., latencies) in the ERP during the 80–180, 170–260, and 300–500 ms after stimulus onset for N100, P200, and P300 components, respectively (Figure 2). We tested for significant differences between target and non-target sub-trials for each channel using a paired *t*-test. Like the ERP analysis at Pz, FDR correction was employed (number of samples *N*_*ch*_ = 64).

### BCI performance evaluation

#### Training a binary classifier and calculating a score

In an offline analysis, we identified the target direction individually for each participant using the six sound directions and 5-fold cross-validation as follows. First, we split the data into five sets. In each cross-validation, four sets were used as training data and the remaining set was the test data. Using the training dataset, we trained a binary classifier to distinguish between target and non-target sub-trials. We used a variant of regularized FDA (Blankertz et al., 2011) as the classification algorithm, which is a supervised learning classifier that is typically used to reduce the dimensionality of data. The method we used was proposed by Gonzalez et al. (2014), where a regularized parameter for FDA is searched for by particle swarm optimization (PSO) (Kennedy, 2011). Although this method can also select EEG channels to be used as feature values, we used all channels without selection.

FDA trained a weight ***w*** that projects input vector ***x*** of D dimensions to scalar *y*. Thus, a hyperplane is obtained as follows:

(2) $$\begin{array}{r} y=w^{T}x \end{array}$$

We determined the ***w*** that maximizes the amount of separation of the data after projection. The objective function to maximize is represented by:

(3) $$\begin{array}{r} J\left(w\right)=\;\frac{{\langle w,\;m_{t}-m_{nt}\rangle }^{2}}{w^{T}S_{w}w+\lambda ||w|{|}^{2}} \end{array}$$

where ***m_t_*** and ***m_nt_*** are averages of the target and non-target sub-trials respectively, and ***m_t_*–*m_nt_*** is the difference between the average of each class. ***S_w_*** is the covariance between classes. Angle brackets denote the inner product and **||*w*||** indicates the Euclidean norm of ***x***. λ is the regularization parameter determined by the PSO. This parameter plays a crucial role as it is needed to prevent imprecisions in the calculation of ***w*** that might appear because of the high dimensionality of the data. When λ is 0, normal FDA is obtained. The parameter λ was determined by 5-fold cross-validation of the PSO in the training data (PSO details are described in Supplementary Materials).

Next, we evaluated the test data using the estimated ***w***. We used the test data as an input dataset by judging which of the target and non-target distributions of training data was closer to the input. Then, we determined a score that represented the distance from a hyperplane (Equation 2). A score of 0 means that the output ***y*** is on the hyperplane. It is recognized as a target if the score is positive, and a non-target if it is negative.

#### Identification of target direction

Because we used six sound directions and wanted to identify the target direction from those directions, we calculated the identification accuracy as the accuracy of identifying the target out of six directions (6-class identification; chance level is about 16.7%). The score described above was calculated for the target estimation. Six trials, one from each direction, were defined as a single set, and the direction in which the score becomes maximum is considered the target direction. We examined the identification accuracy for a single trial and averaged trials for *n* times (*n* = 2, 3, …, 10). When performing the averaging, the score of a single trial was averaged for each direction, and the direction in which the average score was the maximum was set as the estimated target direction.

Thus, we calculated identification accuracy for each cross-validation repetition and obtained averaged identification accuracy across repetitions and participants. To examine the effect of SOA on the identification accuracy, we conducted a three-way analysis of variance (ANOVA) with factors of SOA, a number of averaged trials, and participant. Differences compared with the 1,100 SOA condition (used in our previous study) were tested using a Turkey-Kramer *post-hoc* test.

#### BCI utility

Information transfer rate (ITR) is commonly used to evaluate BCI performance (Wolpaw and Wolpaw, 2012). However, ITR does not consider error correction if it is used in an offline analysis. Furthermore, by its definition, ITR becomes higher when the SOA is small, even if the accuracy (identification accuracy) is low (around 50%). In the present study, we evaluated performance using BCI utility (Dal Seno et al., 2010). The definition of the BCI utility *U* is as follows:

(4) $$U=\{\begin{array}{c} \frac{\left(2P/100-1\right){\text{log}}_{\text{2}}\left(N-1\right)}{c}\;\left(P\geq 50\right)\\ 0\;\left(P<50\right) \end{array}$$

where *P* is the identification accuracy (%) and *N* is the number of directions. Denominator *c* is the duration of a single trial and defined by *c* = *nNt*, where *n* is a number of averaged trials and *t* is the SOA. In contrast to the ITR, this measure takes into account error correction. If the identification accuracy is < 50%, BCI utility is zero because the accumulated probability of detecting the correct direction becomes very small and then error correction is always necessary. Additionally, BCI utility and ITR become equal when the identification accuracy is 100%.

## Results

### Localization-accuracy check

The localization-accuracy check was conducted to ensure that participants could localize the virtual sound accurately without any SOA manipulation (i.e., unshortened SOAs). In this check, all participants reported the virtual sound direction correctly. The localization accuracy was 99.0% averaged across all SOA conditions and all eight participants. The result for the worst SOA condition was 91.7%. We thus confirmed that virtual sounds generated using out-of-head sound localization that was tuned for each participant was well localized for each direction.

### Behavioral performance

We calculated averaged recognition accuracies for all directions across the eight participants in the button-press task, as shown in Table 1.

**Table 1:** Averaged button-press accuracy for eight participants.

**SOA (ms)**	**200**	**300**	**400**	**500**	**600**	**700**	**800**	**1,100**
Accuracy (%)	86.9	90.8	89.9	88.0	92.3	95.3	95.5	96.4
(± SD^a^)	(± 5.0)	(± 8.0)	(± 5.1)	(± 6.2)	(± 5.5)	(± 3.3)	(± 4.2)	(± 4.1)

a *SD, standard deviation over directions*.

Recognition accuracies in all SOA conditions were over 85%, suggesting no or little differences in recognition performance across experimental days. The highest accuracy rate was 96.4% for the 1,100-ms SOA. The lowest result was obtained for the 200-ms SOA (86.9%).

### ERP differences

The results for the averaged ERP time course at channel Pz from 0 to 1,000 ms are shown in Figure 2. We observed that for all but the 200-ms SOA condition, the target and non-target ERPs differed significantly around 400 ms after stimulus onset (*p* < 0.05, FDR corrected). For the 200-ms condition, a paired *t*-test showed a significant difference when correction was not applied (*p* < 0.05, uncorrected).

**Figure 3:**
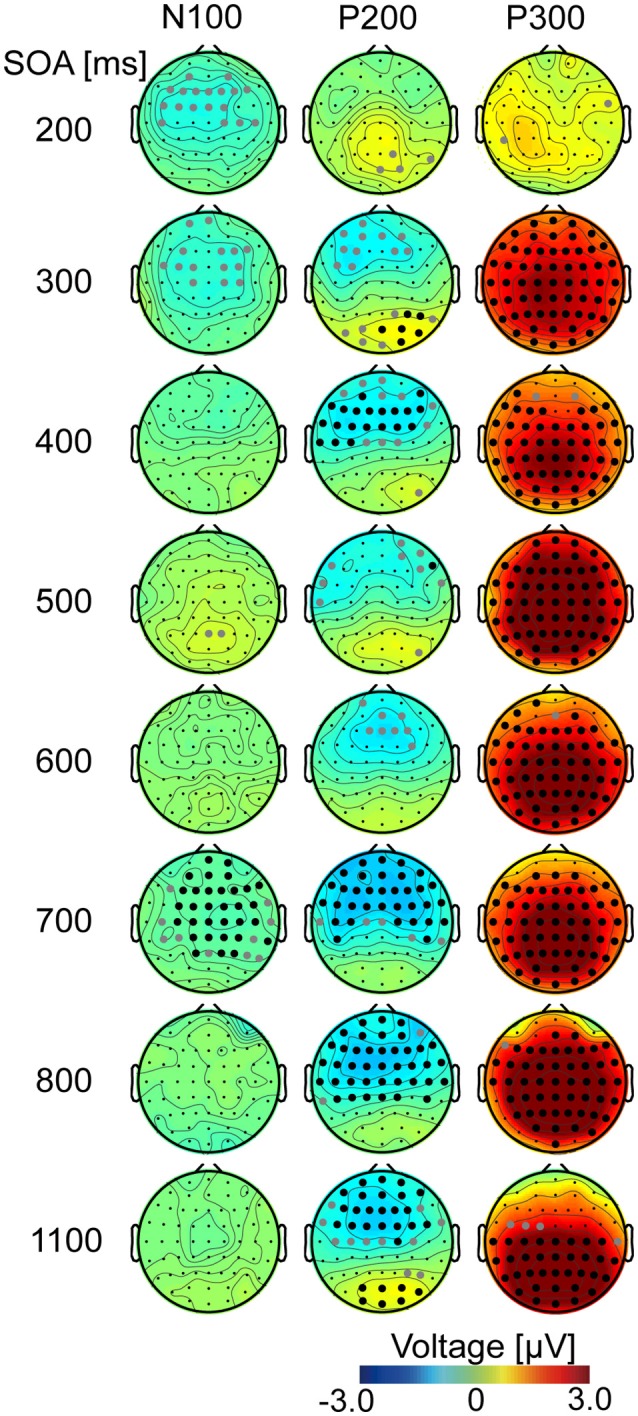
Topography of ERP difference (target – non-target). Differences in ERPs were calculated from averaged EEG data for all participants. Each row represents one of the eight SOA conditions and shows difference maps at each latency for N100, P200, and P300 (Supplementary Table 1). White and gray filled circles represent channels that showed statistically significant differences (*p* < 0.05) with and without FDR correction, respectively.

To examine differences in the EEG waveforms, EEG data for N100, P200, and P300 were averaged across participants in every SOA condition for each channel (Figure 3). The latency of each ERP is listed in Supplementary Table 1.

At the N100 time point (Figure 3, left), channel showed a significant difference between target and non-target (with FDR corrected) only in the 700-ms SOA condition. In contrast, at the P200 time point (Figure 3, middle), some channels were significantly different (*p* < 0.05, FDR corrected), except when the SOA was 200, 300, or 600 ms. These changes had negative values for the frontal areas, suggesting a decreasing P200 component. At the P300 time point (Figure 3, right), ERPs in target and non-target sub-trials were significantly different for the central and posterior channels (*p* < 0.05, FDR corrected). This was not observed in the 200-ms SOA condition. The amplitude was larger in the target sub-trials than in the non-target sub-trials because the observed ERP differences were positive, which probably reflects P300. No channel showed significance at any time for the 200-ms SOA.

To determine if P300 amplitude was related to direction identification, we examined the correlations between peak differences of P300 amplitude between target and non-target sub-trials at three representative channels (Fz, Cz, and Pz) and the single-trial identification accuracy for each SOA. Results showed strong positive correlations between P300 amplitude and identification accuracy (*r* = 0.88, 0.91, and 0.83 for each respective channel; all *ps* < 0.05; *n* = 9 samples). Similarly, we also tested whether amplitude difference between target and non-target sub-trials before P300 (minimum value during 150–300 ms) negatively correlated with single-trial identification accuracies for frontal and central channels (Fz and Cz). Analysis revealed no significant correlations (*r* = −0.45 and 0.10 for the Fz and Cz, respectively; *p* > 0.05), but found a tendency toward a negative correlation at the Fz channel.

### Identification accuracy and BCI utility

**Figure 4:**
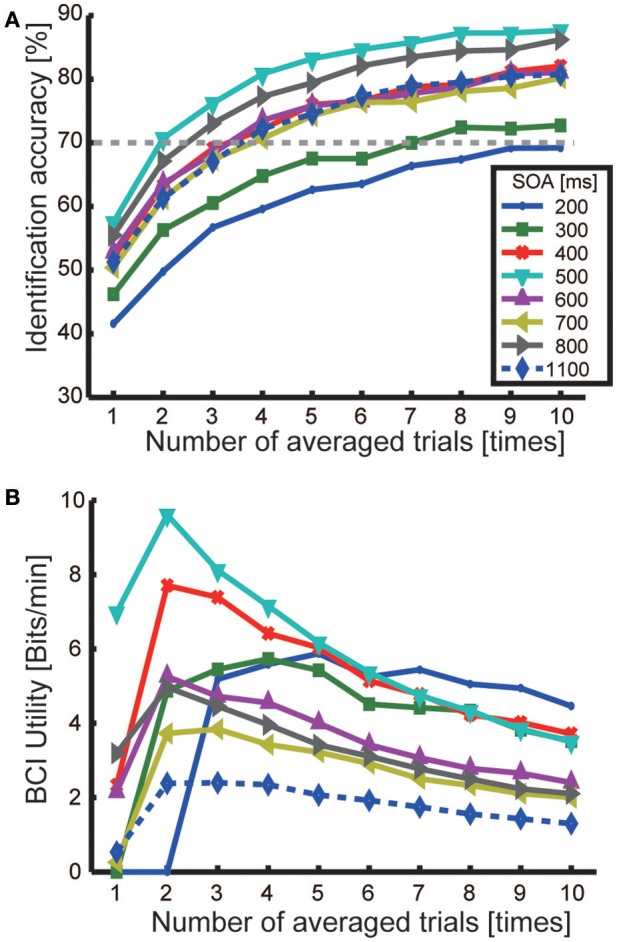
Identification accuracy of target direction and BCI utility. **(A)** Identification accuracies. Each line represents the average accuracy across participants in each SOA condition as a function of the number of averaged trials (1–10 times). **(B)** BCI utility for each SOA condition as a function of the number of averaged trials.

The average accuracy in identifying target direction across participants was determined for each SOA condition (Figure 4A). Averaging-induced increases in identification accuracy can be seen in all SOA conditions. Identification accuracy reached about 80% in many SOA conditions. Accuracies for the 200-ms and 300-ms conditions were relatively low (around 70% at maximum) compared with the other SOA conditions. When SOA was 400–1,100 ms, identification accuracies became 70% when averaging four trials and rose to around 80% when averaging more than nine trials. The highest accuracies were found for the 500 and 800-ms SOAs. The highest accuracies were achieved for the 500-ms (87.7% at maximum, averaged 10 times) and 800-ms (86.2% for the, averaged 10 times) SOAs. A three-way ANOVA revealed a main effect of SOA [*F*_(7, 719)_ = 18.69, *p* < 0.001]. Turkey-Kramer post hoc tests showed that performance for the 500-ms SOA was significantly higher (*p* < 0.002) than that for an 1,100-ms SOA, while those for the 200-ms and 300-ms SOAs were significantly lower (*p* < 0.001 and *p* < 0.02, respectively).

Analysis with the BCI utility *U* revealed a maximal *U* of 9.5 bits/min for the 500-ms SOA when averaging twice. The *U* for the 400-ms SOA condition was also high (7.2 bits/min).

In summary, even when the SOA was shortened by 400 ms, performance accuracy was higher or almost the same as were observed with the 1,100-ms SOA. As a result, the BCI utility increased to 9.5 bit/min.

## Discussion

In this study, we examined the impact of shortening the interval of the stimuli presented (SOA) in an auditory BCI that estimates user intention during a virtual sound-listening task. To this end, we analyzed both behavioral and EEG data (ERP, identification accuracy, and BCI utility).

The results of the behavioral experiment showed that participants obtained target recognition accuracies greater than 85% in all SOA conditions. This indicates that they were able to recognize the target direction and complete the required task. In the EEG experiment, significant ERP differences were found between target and non-target sub-trials for 300–1,100-ms SOAs, and higher identification accuracy and BCI utility were observed when the SOA was shortened to 400 and 500 ms. Thus, compared with the 1,100-ms SOA used in previous research (Nambu et al., 2013), we confirm that shortening the SOA can indeed improve performance in an auditory BCI using virtual sounds.

### Target recognition ability

Here, we calculated three different measures to examine the feasibility of shorter SOAs in an auditory BCI using virtual sounds.

First, target-recognition performance was assessed as a behavioral measure. It is important to understand whether SOAs used in the experiment are long enough for participants to recognize the target stimuli in an oddball paradigm. Yet, in previous studies of auditory BCIs using spatial sound information (Gao et al., 2011; Käthner et al., 2013; Nambu et al., 2013; Simon et al., 2014), the behavioral performance was not considered. Only one study using loudspeakers (Schreuder et al., 2010) examined recognition performance for eight spatial directions of sound (condition Cr). However, whether their participants could recognize target direction with a very short SOA is not clear because they used a ~2,000-ms SOA, which was much longer than those generally used in BCI experiments. The current results of our behavioral experiment showed that target recognition accuracy was over 85% in all SOA conditions, indicating that participants could recognize the target direction fairly easily in a spatial auditory BCI paradigm even when the SOA was very short (200 ms). Thus, our results highlight the effectiveness of very short SOAs (down to 200 ms) in target recognition.

We should note a methodological limitation when evaluating behavioral performance in short SOA conditions (200 and 300 ms). To evaluate recognition performance, we counted the responses during the 150–600 ms after stimulus onset for short SOA conditions (see 2.4.2). For 200 and 300-ms SOAs, this duration overlaps with non-target stimuli before and after the target stimulus. Thus, a response to the non-target stimulus before or after the target stimulus could potentially be wrongly considered as a correct response to the target. However, we think that such misrecognition of responses is unlikely because the responses (mean reaction times) were similar across SOA conditions (Supplementary Table 2).

The high target recognition that we observed in the behavioral task was likely maintained during the EEG experiment because the cognitive processing required for perceiving virtual sounds was virtually the same between the two experiments. The only difference between experiments was how the participants reported a sound from the target detection: in the EEG experiment they silently counted the number of times that they occurred, while in the behavioral experiment they pressed a button as soon as they heard one. In fact, errors during silent counting were <1.5% for all SOA conditions (Supplementary Table 3), supporting successful target recognition. Thus, we conclude that participants were able to recognize the target direction in the EEG experiment when the SOAs were very short.

### Relationship between behaviors, evoked potentials, and identification

Now we consider the relationship between identification accuracy and the other factors. The ERP results (Figures 2, 3) showed two types of differences between target and non-target sub-trials. One was the strong P300 component in the target sub-trials. This was observed over many channels for all but the 200-ms SOA. The lack of a strong P300 component at very short SOAs is in line with previous ERP studies (see Polich, 2007 for a review). For example, P300 was reported to be small when target-to-target intervals for either auditory or visual stimuli were 1 s (Gonsalvez and Polich, 2002). In the current study, the average target-to-target interval for the 200-ms SOA was 1.2 s, and we saw similarly low small P300 amplitudes. Overall, the correlation analyses revealed a strong correlation between P300 amplitude and identification accuracy, suggesting that this ERP is related to identification of direction, which is associated with attentional allocation processing (Polich, 2007).

The other difference that we observed was in a negative component. A previous study (Allison and Pineda, 2006) examined the effects of short SOA on ERPs in a visual BCI setting and suggested that in addition to the three ERPs examined here, N200 is also influenced by SOA. Similar results have also been observed in auditory BCIs (Schreuder et al., 2010; Halder et al., 2013; Nambu et al., 2013). In line with these findings, we observed differences in negative components 150–300 ms after stimulus onset, primarily distributed in the frontal and central channels (Figure 3). We found a tendency for the amplitude in this negative component to negatively correlate with identification accuracy for the Fz channel (but not significantly). This might thus reflect a decrease in P200 or N200 components in the frontal/central areas.

Thus, P300 and negative components (decrease in P200/N200) were consistently observed even when the SOA was shortened. Based on our results, P300 is a strong indicator of identification accuracy, and frontal negative components may also be related.

### Toward optimization of the SOA

The best identification accuracy that we observed and the best BCI utility were achieved at a 500-ms SOA (Figure 4). We compared our current results for seven different SOAs (ranging from 200 to 800 ms) with those from the 1,100-ms SOA used in our previous study (Nambu et al., 2013). The results showed that identification accuracy for the 400–800 ms SOA conditions were higher or about equal to the accuracy at an SOA of 1,100 ms (significant improvement was found for the 500-ms SOA). Using BCI utility, we found that the maximum BCI performance for all SOA conditions were improved compared with those for the 1,100-ms SOA. This suggests that shortening the SOA is crucial for improving BCI performance.

Our results extend the findings from past studies using virtual sound (Gao et al., 2011; Käthner et al., 2013). Gao et al. (2011) examined a BCI system using virtual sound from five directions. They used randomized SOAs from 300 to 500 ms and obtained ~40% accuracy (binary classification) in single trial and more than 80% accuracy with 10-trial averaging. In our current study, accuracies were relatively higher; 200 and 500-ms SOAs resulted in single-trial accuracies of 41.6 and 57.5%, respectively. This discrepancy might have resulted from differences in the measurement environment when creating the virtual sound source (they used an HRTF database), and the use of a support vector machine as the classifier. For that reason, we suggest the effectiveness of virtual sound generated by individual HRTFs and the FDA classifier used in this study. In another study (Käthner et al., 2013), Käthner and colleagues used SOAs from 200 to 600 ms and obtained the highest identification accuracy for the 600-ms SOA (560 ms ISI) and the best ITR for the 440-ms SOA (2.76 bits/min). These were in line with our results in which better performances were obtained using 400 and 500 ms SOAs. Furthermore, we also observed higher BCI utility, which was likely because of shortening the SOA, reducing the number of averaged trials, better direction recognition using individual HRTF, and the different classifier algorithm (regularized FDA).

Another study (Schreuder et al., 2010) examined shortened SOAs using loudspeakers for eight directions. They obtained about a 30–60% identification accuracy at an SOA of 215 ms in a single trial using five participants. Our results were similar to these. This previous study used five frontal directions (45° apart) and loudspeakers. Thus, the task used in the previous study was much simpler than the task used in our study. However, identification accuracies did not show large differences. From this, our results also suggest that for auditory BCIs, shortening SOAs in combination with virtual sounds that employ individual HRTFs is effective.

Very short SOA has been shown to be feasible for visual BCIs (Farwell and Donchin, 1988; Sellers et al., 2006; McFarland et al., 2011; Lu et al., 2013). In auditory BCIs, studies using loudspeakers have used very short SOAs of around 200 ms (Schreuder et al., 2010, 2011) and another study suggests that SOAs can be shortened to around 200 ms in a simple auditory setting without spatial information (Höhne and Tangermann, 2012). In contrast to these findings, our results indicate that auditory BCIs using *virtual sound* can be optimal for anyone when SOAs are between 400 and 500 ms. Although our results showed that an SOA of 200 ms resulted in low performance on average, this does not mean that a 200-ms SOA is inappropriate for use in auditory BCIs using virtual sounds. Rather, our results indicate that individual differences in the ability to identify the target location at 200-ms SOA were large. Because some participants did quite well at this very short SOA (more than 80% after averaging; Supplementary Figure 1), the appropriateness of a 200-ms SOA should be evaluated on a case-by-case basis.

We also confirmed from our behavioral experiment results that the recognition accuracy at an SOA of 200 ms was low compared with the other SOA conditions even though the recognition accuracy was over 85%. (Table 1). This could be related to the low identification accuracy at an SOA of 200 ms. For that reason, we suggest that identification accuracy can be improved when conducting EEG by training participants on the tasks before beginning the experiments. The present study focused on recognition of sound using spatial information. To this end, we used the same white noise stimuli for all directions. The virtual sounds in the current setting were 60° apart. This resolution is enough for participants to recognize the sound correctly. Although virtual sounds with spatial resolutions of <15° are possible (Shimada and Hayashi, 1995; Middlebrooks, 1999), increasing the resolution beyond 60° will likely result in increases in recognition errors. Performance could be also improved by adopting different sound patterns for each direction, for example, different tones (Schreuder et al., 2010, 2011; Käthner et al., 2013), or natural stimuli (Simon et al., 2014). We intend to study this in the future.

## Conclusion

In this study, we examined the impact of shortening SOAs on an auditory BCI using virtual sounds. By assessing both behavioral performance and brain activity data in offline analysis, we confirmed that SOAs can be shortened and the best improvements in BCI utility could be achieved with a 500-ms SOA. Considering individual differences, good identification accuracies can likely be achieved for many people even at an SOA of 200 ms. Thus, shortening the SOA is an effective means for improving auditory BCIs that use virtual sounds. In the future, we will consider shortening SOAs in online practical settings.

## Author contributions

MS, YH, IN, and YW: Conceived and designed the study; MS and YH: Performed the experiments; MS, YH, and IN: Analyzed the data; AG, SY, HH, and YW: contributed analysis tool; MS: Wrote the first draft of the manuscript; MS, IN, YT, and YW: Revised the paper.

### Conflict of interest statement

The authors declare that the research was conducted in the absence of any commercial or financial relationships that could be construed as a potential conflict of interest.

## Footnotes

^1^www.biosemi.com.
