# Supplementary material for: Improving the Performance of an Auditory Brain-Computer Interface Using Virtual Sound Sources by Shortening Stimulus Onset Asynchrony
Source: Front Neurosci. 2018 Feb 27;12:108. doi: 10.3389/fnins.2018.00108 (PMC5835086; doi:10.3389/fnins.2018.00108)
Supplement: Supplementary file 1 [file Presentation1.pdf]

Supplementary Materials

Improving the Performance of an Auditory Brain-Computer Interface using Virtual Sound Sources by Shortening Stimulus Onset Asynchrony

Miho Sugi, Yutaka Hagimoto, Isao Nambu*, Alejandro Gonzalez, Yoshinori Takei, Shohei Yano, Haruhide Hokari, Yasuhiro Wada

*** Correspondence: Isao Nambu: inambu (at) vos.nagaokaut.ac.jp**

1. Particle Swarm Optimization

To determine the regularization parameter λ for FDA, we used PSO (Gonzalez et al., 2014; Kennedy 2011). PSO is a probability-based method for optimization. When the objective function to be searched is given, a group of particles share information with each other and move around in the search space until the global solution reaches the desired fitness. A particle in PSO can be expressed as a vector using p_i^t for position of the i-th particle and v_i^t for its velocity at certain iteration t as stated below:

$$p_i^t = p_i^{t-1} + v_i^t, \tag{S1}$$

$$v_i^t = av_i^{t-1} + c_1\eta_1(p_i - p_i^{t-1}) + c_2\eta_2(g - p_i^{t-1}). \tag{S2}$$

In the calculation of velocity, p_i is the best individual solution for a single particle and $g \in [-1, 1]$ is the best solution found for all the particle system; c_1 and c_2 are constants and both are set to 2 in this study; η_1 and η_2 are random numbers. The inertia parameter is defined by a. Also, we used following equation to calculate the regularization parameter:

$$\lambda = 10^g. \tag{S3}$$

2. Identification accuracies for individuals in the 200 ms SOA.

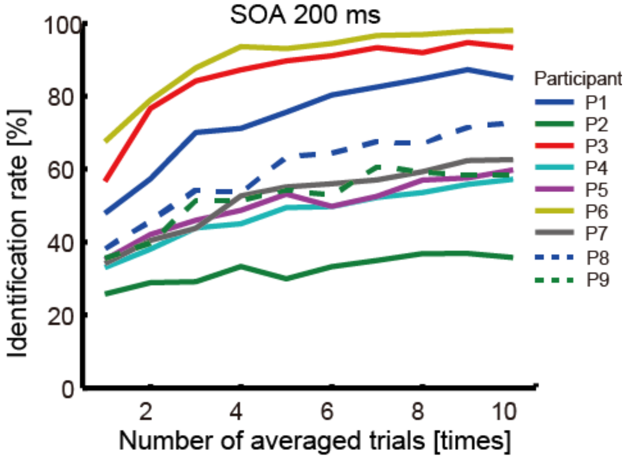

Supplementary Fig 1. Identification accuracy for each participant in the 200-ms SOA condition. Each line represents the accuracy for one participant.

3. ERP latencies

Supplementary Table 1. Averaged latencies for N100, P200, and P300.

**SOA** [ms]		200	300	400	500	600	700	800	1100
**Latency** [ms]	N100	126	110	99	95	95	91	91	83
	P200	260	217	197	193	193	193	193	193
	P300	450	374	370	382	374	359	367	367

4. Reaction time

Supplementary Table 2. Averaged Reaction Time.

**SOA** [ms]	200	300	400	500	600	700	800	1100
**RT** [ms] (± SD[a])	387 (± 22)	397 (± 31)	411 (± 28)	425 (± 35)	438 (± 36)	445 (± 58)	453 (± 66)	505 (± 97)

[a] SD: standard deviation across participants

5. Counting errors in the EEG experiments

Supplementary Table 3. Averaged counting errors in the EEG experiment.

**SOA** [ms]	200	300	400[b]	500[b]	600	700	800	1100
**Counting Error** [%] (± SD[a])	1.2 (± 1.0)	1.4 (± 1.6)	1.0 (± 1.2)	0.9 (± 1.0)	1.2 (± 0.8)	1.4 (± 1.5)	0.9 (± 0.9)	1.5 (± 0.9)

[a] SD: standard deviation across participants
[b] Data was calculated from six out of eight participants because of missing data.
